# Review on melanosis coli and anthraquinone-containing traditional Chinese herbs that cause melanosis coli

**DOI:** 10.3389/fphar.2023.1160480

**Published:** 2023-05-04

**Authors:** Ruiyuan Zhang, Cai Huang, Fan Wu, Ke Fang, Shujun Jiang, Yan Zhao, Guang Chen, Ruolan Dong

**Affiliations:** ^1^ Department of Integrated Traditional Chinese and Western Medicine, Tongji Hospital, Tongji Medical College, Huazhong University of Science and Technology, Wuhan, Hubei, China; ^2^ Grade 2019 of Integrated Traditional Chinese and Western Clinical Medicine, Second Clinical School, Tongji Medical College, Huazhong University of Science and Technology, Wuhan, Hubei, China

**Keywords:** melanosis coli, anthraquinones, traditional Chinese herbs, constipation, laxative

## Abstract

**Backgrounds:** The incidence of melanosis coli (MC) has gradually increased annually, attracting significant attention and efforts into this field. A potential risk for MC is the long-term use of anthraquinone laxatives in patients with constipation. Most traditional cathartic drugs are made from herbs containing anthraquinone compounds. This review aims to provide guidance for the application of traditional Chinese herbs containing anthraquinones for physicians and researchers.

**Materials and methods:** We reviewed risk factors and pathogenesis of MC, and natural anthraquinones isolated from TCM herbs. We searched Pubmed and CNKI databases for literature related to MC with keywords such as“traditional Chinese medicine”, “Chinese herbs”, “anthraquinones”, and “melanosis coli”. The literature is current to January 2023 when the searches were last completed. After the literature retrieval, the TCM herbs containing anthraquinones (including component identification and anthraquinone content determination) applied in clinical were selected. According to the collected evidence, we provide a list of herbs containing anthraquinones that could cause MC.

**Results:** We identified 20 herbs belonging to 7 families represented by Polygonaceae, Fabaceae, Rhamnaceae, and Rubiaceae, which may play a role in the pathogenesis of MC. Among these, the herbs most commonly used include Dahuang (Rhei Radix et Rhizome), Heshouwu (Radix Polygoni Multiflori), Huzhang (Rhizoma Polygoni Cuspidati), Juemingzi (Semen Cassiae), Luhui (Aloe) and Qiancao (Rubiae Radix et Rhizoma).

**Conclusion:** Due to a lack of awareness of the chemical composition of TCM herbs, many patients with constipation and even some TCM physicians take cathartic herbal remedies containing abundant anthraquinones to relieve defecation disturbances, resulting in long-term dependence on these herbs, which is potentially associated with most cases of MC. When such treatments are prescribed, TCM physicians should avoid long-term use in large doses to reduce their harm on colonic health. Individuals who take healthcare products containing these herbs should also be under the supervision of a doctor.

## 1 Melanosis coli

### 1.1 Introduction to melanosis coli

Melanosis Coli (MC) is a reversible, non-inflammatory, and non-precancerous disease characterized by brownish pigmentation of the colonic mucosa. Billiard noted this colonic pigmentation in 1825 for the first time, and Cruveilhier described it for the first time in 1829. In 1857, Virchow named the condition ‘Melanosis Coli’, which was subsequently defined as a benign condition.

The characteristic image of MC is granular or reticular brownish pigmentation distributed in part or the whole colonic mucosa under endoscope (Grilo et al., 2014). Histological observation of colon pigmentation under the light microscope shows that epithelial cells and different levels of macrophage deposition are distinguished in the mucosa lamina propria. Colonic mucosal biopsies examined by electron microscopy show abnormalities of absorptive epithelial cells, apoptosis of colonic epithelial cells, and phagocytosis of apoptotic bodies by macrophages in the epithelium. On the surface of absorptive cells, the microvilli become less and even disappear. Macrophages migrate towards the lamina propria, where lipofuscin was formed under intracellular degradation of the apoptotic bodies. Apart from pigment-laden macrophages, plasma cells and degenerated nerves are also present in the lamina propria. (Balázs, 1986; Walker et al., 1993).

There are no individual symptoms or physical signs in patients with MC. The most relevant symptom is constipation, which is one of the etiologies of MC. The morbidity of MC is between 0.82% and 1.13%. MC tends to occur in individuals who rely on anthraquinone laxatives to relieve constipation and individuals who use laxatives as weight-reducing drugs, beauty products, and health products. Approximately 95% of MC patients have a medication history of taking anthraquinone laxatives for a prolonged time, especially in females. Studies revealed that males in their 60s and females in their 50s were more likely to suffer from MC. (Yang et al., 2020b). A multicenter study based on 6,090 cases in China concluded that MC detection rates increased with age (Wang et al., 2018). The colonic polyp is the most common concomitant disease of MC in the Chinese population. (Wang et al., 2018).

### 1.2 Etiology and risk factors of melanosis coli

#### 1.2.1 Anthraquinone laxatives

**FIGURE 1:**
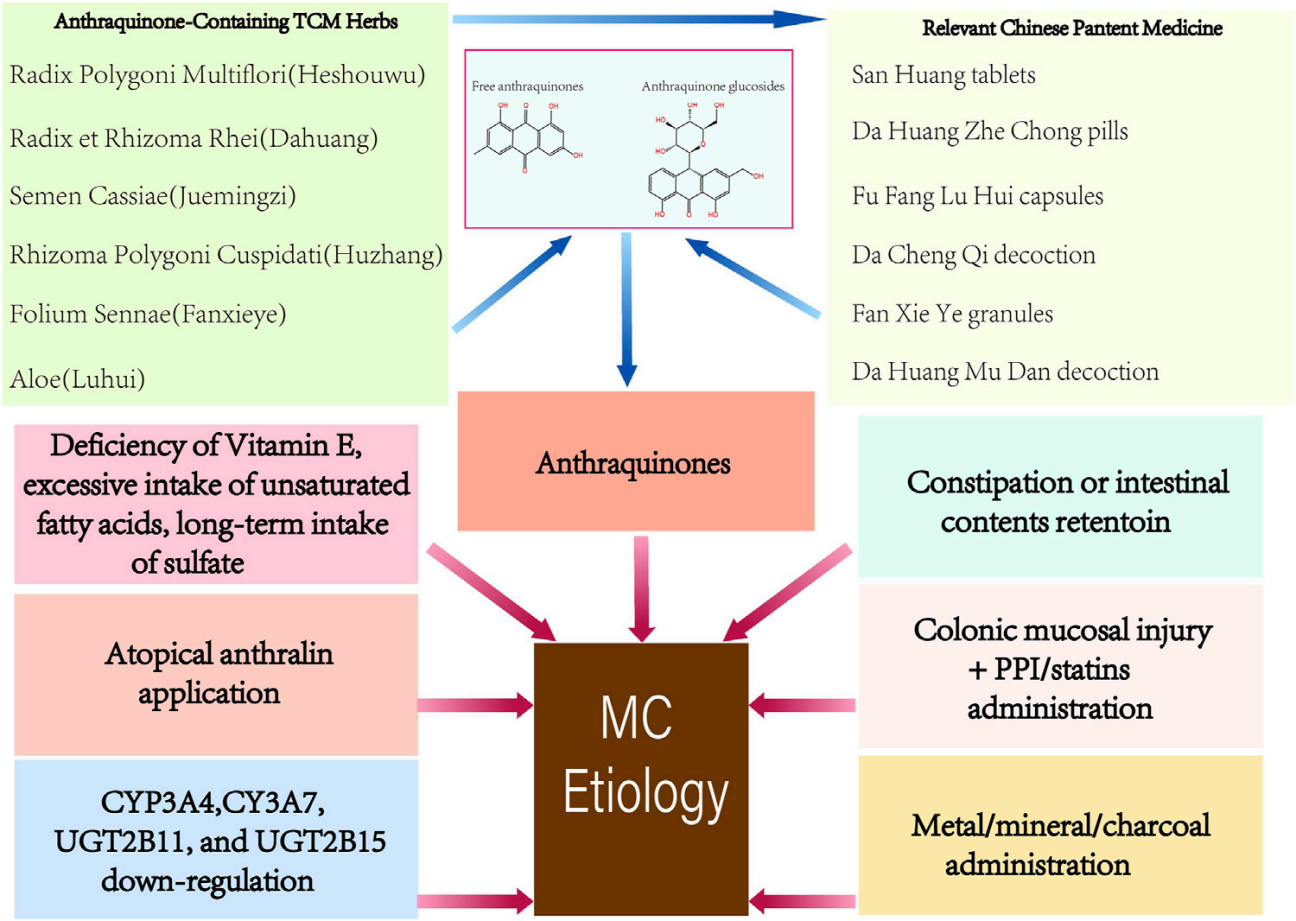
Etiology of Melanosis Coli.

**FIGURE 2:**
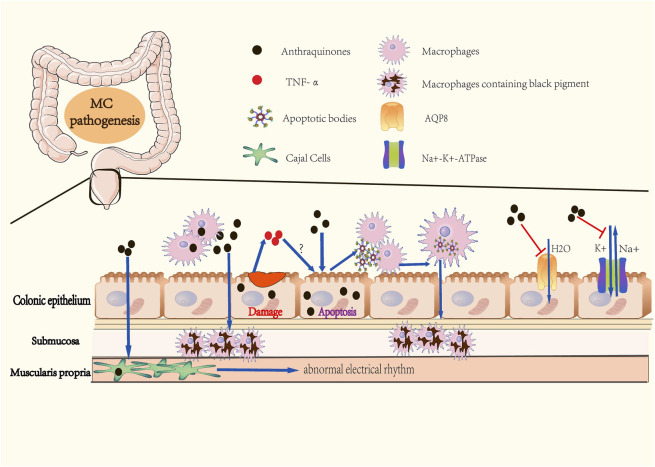
Pathogenesis of Melanosis Coli. **(A)** Colonic epitheliums absorb anthraquinones in the colonic cavity. **(B)** Macrophages directly intake anthraquinones, then migrate to submucosa. **(C)** Anthraquinones cause abnormal apoptosis of colonic epithelial cells, followed by macrophages phagocytizing apoptotic bodies. This step may be facilitated by TNF-α release caused by anthraquinones-induced epithelial damage. Macrophages containing apoptotic bodies migrate to submucosa and form black pigments. **(D)** Anthraquinones retain water in the colonic cavity by inhibiting the activities of Na^+^-K^+^-ATPase and AQP8. Anthraquinones also contribute to abnormal electrial rhythm of Cajal cells. These mechanisms promote dependency on anthraquinones in individuals with constipation.

The abuse of anthraquinones was the first identified and the primary cause of MC (Figure 1). As contact cathartics, anthraquinones help relieve constipation by promoting bowel motility and reabsorption of water in the colonic mucosa. However, its side effects and harm to the human body are evident with long-term administration. Scientists have proposed several theories on how anthraquinones contribute to MC (Wan et al., 2008). The initial theory is that the colon epithelium absorbs anthranoid combinations because most research correlates black pigment with anthraquinones metabolites due to their similarities in color. Hoshi et al. assumed that the pigmentation in macrophages resulted from their direct uptake of anthraquinones (Hoshi et al., 1996), (Figure 2). With the help of advanced technology, it was confirmed that absorbed anthranoid free radicals exist in the melanosis tissue (Krbavcic et al., 1998), (Figure 2). Furthermore, some researchers have proposed that anthraquinones could activate enzymes that involve in melanin pigmentation and intestinal dysfunction (Wan et al., 2008).

#### 1.2.2 Constipation or retention of intestinal contents

Many researchers have focused on constipation, the most common symptom of MC, to identify a more reasonable explanation of the onset of colonic mucous melanosis (Figure 1). Instead of abandoning the view of the vital role of anthraquinones in MC, it has been proposed that chronic use of anthraquinones masks the underlying history of refractory constipation in patients. There are two main hypotheses about how constipation leads to MC. One theory is that the colon absorbs brownish pigment granules synthesized by intestinal microorganisms; the other is that digestive enzymes decompose proteins originating from incompletely digested food into peptides and amino acids, which are turned into pigment granules by submucosal enzymes, and such granules are absorbed by the colon mucosa eventually (Wan et al., 2008).

#### 1.2.3 Other causes

Apart from anthraquinones, anthralin for topical application (Lestina, 2001), bisacodyl (Mennecier and Vergeau, 2004), proton-pump inhibitor (PPI) (Dore et al., 2014), statins (Dore et al., 2014), ipecac (Johnson et al., 1991), and bamboo leaf extract (Iseki et al., 1998) have been reported to cause MC lesions (Figure 1). Deficiency of vitamin E, excessive intake of unsaturated fatty acids, oxidative damage (Wilberts et al., 2015), and drinking sulfate-contaminated water (Rodríguez-Gómez et al., 2021) for a prolonged time have also been reported as possible factors associated with MC (Figure 1). Chronic oral administration or respiratory inhalation of metallic elements (Cha et al., 2009) or mineral substances (Kim et al., 2011) is considered to be responsible for intestinal melanosis (Figure 1). Progress in genetic polymorphisms research shows that the cytochrome P450-related genes CYP3A4, CYP3A7, UGT2B11, and UGT2B15 were significantly downregulated in MC (Li et al., 2015), (Figure 1).

### 1.3 Pathogenesis of melanosis coli

In 1975, Steer and Colin-Jones determined that the lysosomal activity and the number of lysosomes in macrophages, Schwann cells, and neurons of the submucosal plexus of the colonic mucosa increased in MC patients with abuse of anthraquinones (Steer and Colin-Jones, 1975). In 1988, Walker et al. observed that daily oral administration of anthraquinones in MC guinea pigs induced transient dose-related apoptosis in colonic epithelial cells (Walker et al., 1988). Research in MC patients by Walker et al. revealed the involvement of apoptotic bodies in the pathogenesis of MC (Walker et al., 1993). They deduced that macrophages possessing phagocytosed apoptotic bodies could cross the fenestrae in the basal lamina, which is similar to those present in the small intestine and skin. Due to existing limitations in both anthraquinone purgatives- and constipation-related theories of the pathogenesis of MC, some scholars changed their focus and developed a theory whereby the increased apoptosis of colonic epithelial cells is the root cause of MC. Byers et al. measured the colonic epithelial apoptosis of 38 MC patients, and proposed that MC is simply a nonspecific manifestation of increased apoptosis of colonic epithelial cells (Byers et al., 1997). In other words, any elements that facilitates the apoptosis of colonic epithelial cells can underlie the etiology of MC. Chen et al. found that rhubarb increased TNF-α levels both in the serum and colon of guinea pigs with MC (Chen J. Y. et al., 2009). Their follow-up research in MC guinea pigs also revealed that there was a positive correlation in the severity of induced MC with the rate of cell apoptosis and serum TNF-α levels (Chen et al., 2011). Based on these findings, the researchers summarized a possible molecular mechanism for the formation of apoptotic bodies. Anthraquinone destroys the colon mucosal barrier and facilitates the release of pro-inflammatory TNF-α, which causes apoptosis of colonic epithelial cells followed by the production of apoptotic bodies, which ultimately results in the formation of phagocytizing apoptotic bodies by macrophages in the mucosal membrane (Figure 2). Regitnig and Denk determined that apoptotic fragments of epithelial cells remained in the epithelium of adenomas, accompanied by a reduction in macrophage levels and an increased expression of Ki-67 and Bcl-2 in adenoma tissue without melanosis (Regitnig and Denk, 2000). The study suggested that low-grade adenomas or tumors might be related to the absence of melanosis in proliferative lesions, and supported the role of apoptosis in the pathogenesis of MC.

### 1.4 Treatment and prevention of melanosis coli

Patients with MC are reported to have an increased risk of colonic hyperplastic polyps (Katsumata et al., 2021), low-grade adenomas (Kassim et al., 2020) and nonspecific ulceration of the distal ileum (Liu et al., 2017). A retrospective study including 718 MC patients and 2,154 healthy people showed that MC is not related to increased diagnosis of colorectal cancer but enhanced polyp detection due to “chromo-endoscopy-like effect” (Abu Baker et al., 2018). A systemic review aiming to determine the effects of anthraquinone laxatives on colorectal cancer has concluded that a history of anthraquinone laxatives use brought an increased trend in colorectal cancer development, although not showing any significance (Lombardi et al., 2020; Lombardi et al., 2022). Therefore, medications or therapeutic interventions for MC are required. The primary therapeutic objective is to normalize the colon mucosa. Anthraquinones should be discontinued immediately after MC diagnosis (Roerig et al., 2010). Remission of MC is reported to occur in 6 months to 1 year after stopping treatment with anthraquinones (Balázs, 1986). It is suggested that any application period of anthraquinones laxatives longer than 1–2 weeks require physician supervision (Younes et al., 2018). Patients with cosmetic or dietary demands should achieve their goals preferentially by modifying their lifestyle rather than by taking medication. Further, patients with constipation should change their lifestyle and switch to alternative cathartics, such as bulk cathartics, prokinetic agents, osmotic laxatives, and lubricating laxatives. Related food supplement business operators should label the recommended doses on their products; medicinal laxatives containing anthraquinones should be only short-term applied in adults, elderly, and adolescents over 12 years (Younes et al., 2018); some licensed drugs applied in other diseases except for constipation should be labeled the existence of the risk of inducing MC under the condition of long-term use.

## 2 Anthraquinones

### 2.1 Introduction to anthraquinones

Anthraquinones belong to the quinones family characterized by the cyclic diketone structure. Depending on whether the mono-anthracene nucleus is combined with other kinds of compounds, they are classified into two main classes, the free anthraquinones and the combined anthraquinones. The primary members of free anthraquinones are anthracene and various reduction products of anthraquinones, such as anthranol and anthranone; the most significant part of combined anthraquinones are anthraquinone glycosides, whose aglycones are free anthraquinones (Malik and Müller, 2016).

Natural anthraquinones are mainly applied as irritant laxatives, whose pharmacokinetic characteristics have been widely studied. The absorption of anthraquinones mainly occurs in the intestine rather than in the stomach, which is attributed to the longer retention time of anthraquinones in the intestinal tract and the increase in anthraquinones ionization associated with pH level (Malik and Müller, 2016). Most anthraquinones are absorbed by intestinal villous epithelial cells via passive diffusion. Therefore, the absorption of anthraquinones typically depends on their physicochemical properties, especially on the chemical structures and lipid solubilities (Malik and Müller, 2016). After absorption, anthraquinones are widely distributed throughout the body, mainly in organs and tissues with a rich blood supply, such as intestines, stomach, liver, lung, kidney, and fat (Wang D. et al., 2021).

Anthraquinone biotransformation occurs primarily in the liver. Some studies (Malik and Müller, 2016) have shown that oral anthraquinone biotransformation also occurs in the intestine, mainly by the intestinal microorganism such as bifidobacterium, *peptostreptococcus*, and *clostridium*. The metabolic pathways of anthraquinones include mainly hydrolysis, glucuronidation, sulfation, methylation/demethylation, hydroxylation/dehydroxylation, oxidation/reduction, acetylation, and esterification, among which hydrolysis, glucuronidation, and sulfation are dominant (Malik and Müller, 2016). It should be noted that anthraquinones are excreted mainly with the urine, bile or feces in the form of its prototype or metabolites. Anthraquinones excreted with bile can be reabsorbed in the intestines and utilized to form hepato-enteric circulation and maintain a relatively constant blood concentration (Wang X. X. et al., 2021).

Anthraquinones laxatives inhibit the absorption of water and sodium in the colon by inhibiting the activities of Na^+^-K^+^-ATPase of colonic epithelial cells (Malik and Müller, 2016), (Figure 2). Another mechanism involves anthracenes-induced downregulation of AQP8 expression in the colon epithelium, thus retaining water in the colonic cavity (Liu et al., 2011), (Figure 2). In addition to increasing the water content of the stool to soften it and make it easier to discharge, anthraquinones can directly stimulate the plexus of the colonic myenteric nerve and facilitate colon peristalsis activity. Research has reported that emodin can disturb the peristaltic rhythm of the colon, which can be attributed to cell membrane damage and energy metabolism disorder resulting from inhibition of the activities of Na^+^-K^+^-ATPase and Ca^2+^-ATPase and over-activation of IP3 and cAMP in Cajal interstitial cells (Peng et al., 2009), (Figure 2).

For patients with constipation and individuals relied on anthraquinone-containing healthcare products, the most noteworthy item is the intestinal toxicity of anthraquinones (Lombardi et al., 2020). Anthraquinone laxatives have been demonstrated to have tumor-inducing potential in animal studies. Exposing to an anthraquinone-containing diet for 480 days induced 25 colorectal cancer in rats; another animal study showed that using anthraquinone laxatives for 112 days developed 4 adenocarcinomas and 5 adenomas in the large intestine of rats (Mohammed et al., 2021). However, a prospective case control study demonstrated that the long-term use of anthraquinone laxatives was not associated with increased risk for colorectal adenoma or colorectal cancer (Nusko et al., 2000). Considering the potential oncogenicity of anthraquinones, the Panel on Food Additives and Nutrient Sources added to Food (ANS) proposed some application suggestions for the use of anthraquinone-containing food supplements, healthcare products, ethicals and herbs. The daily dose of short-term application of anthraquinone laxative in adults to relieve constipation should be controlled within 30 mg/d and 2–3 times per week (Younes et al., 2018). In addition, products containing anthraquinones should be marked with special symbols, to make medical workers and purchasers realize that the using term, recommended dose, intestinal toxicity, contraindications are several key items for regular use of anthraquinones. Especially for people with other risk factors of colorectal cancer, careful use of anthraquinones might help mitigate the risks of developing colorectal cancer. Either healthcare supplement producers or medical pharmaceutical companies, they all should perform safety experiments of anthraquinone-containing production and submit their maximum recommended doses to food and drug administration. It is a pity that TCM herbal products on the market in China are rarely marked with potential toxicity or contraindications. Therefore, users need to carefully check whether the compositions of these drugs or food supplements include any of the herbs listed here in order to use them with caution. There is still a plenty room for improvement in the food and drug administration.

### 2.2 Common anthraquinones present in TCM herbs

Anthraquinones are widely distributed in Chinese medicine, but the types and contents differ. For Dahuang (Rhei Radix Et Rhizoma), Huzhang (Rhizoma Polygoni Cuspidati), Heshowu (Radix Polygoni Multiflori) and Juemingzi (Semen Cassiae), anthraquinones are the main pharmacological compounds in them. However, anthraquinones in most other herbs are discovered unexpectedly, for that anthraquinones is not a compelling reason to explain their traditional use. Currently, emodin, aloe-emodin and aloin are the most thoroughly studied natural anthraquinone. Dahuang was the first anthraquinone-containing Chinese herb attracting widespread attention because it has been widely used in TCM in all ages, and its effective cathartic function and antimicrobial activity corresponds to its conventional use. Therefore, its main active composition, emodin and related derivatives, have also raised most attention of scholars (Yang et al., 2020a). Till now, research on medicine leading to MC has always been placed emphasis on emodin or aloe emodin, as well as related prescriptions and herbal extracts. Nevertheless, we cannot indiscreetly draw a conclusion that emodin is the most abundant anthraquinone extracted from TCM herbs and it has the most potential to cause MC. In order to provide a reference for rational herbal use in clinic, we briefly summarized the anthraquinones extracted from TCM herbs. It needs to be clarified that the MC lesions are caused by the combined effects of these anthraquinone compounds rather than single one.

**FIGURE 3:**
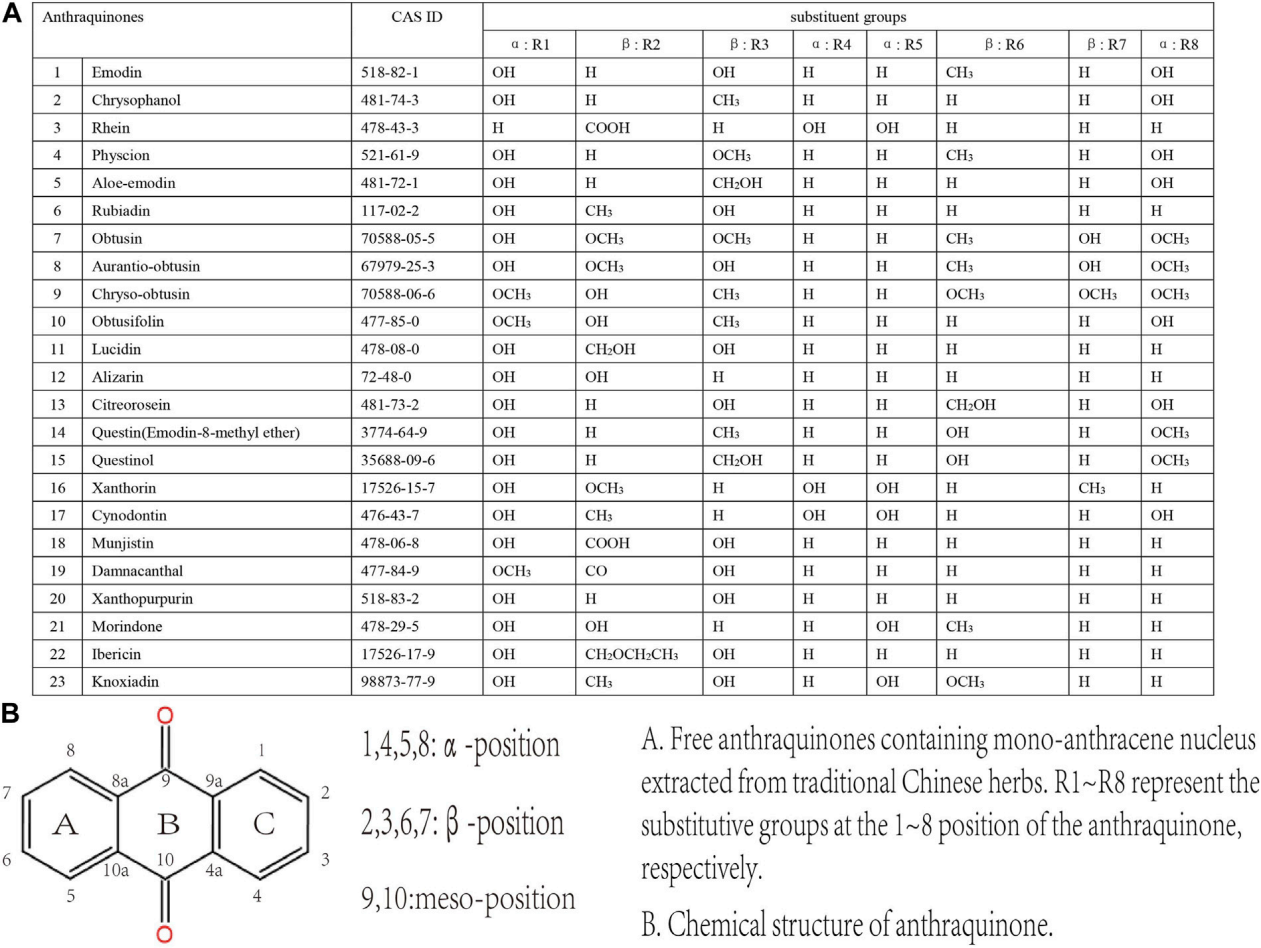
Free anthraquinones containing mono-anthracene nucleus extracted from TCM herbs.

**FIGURE 4:**
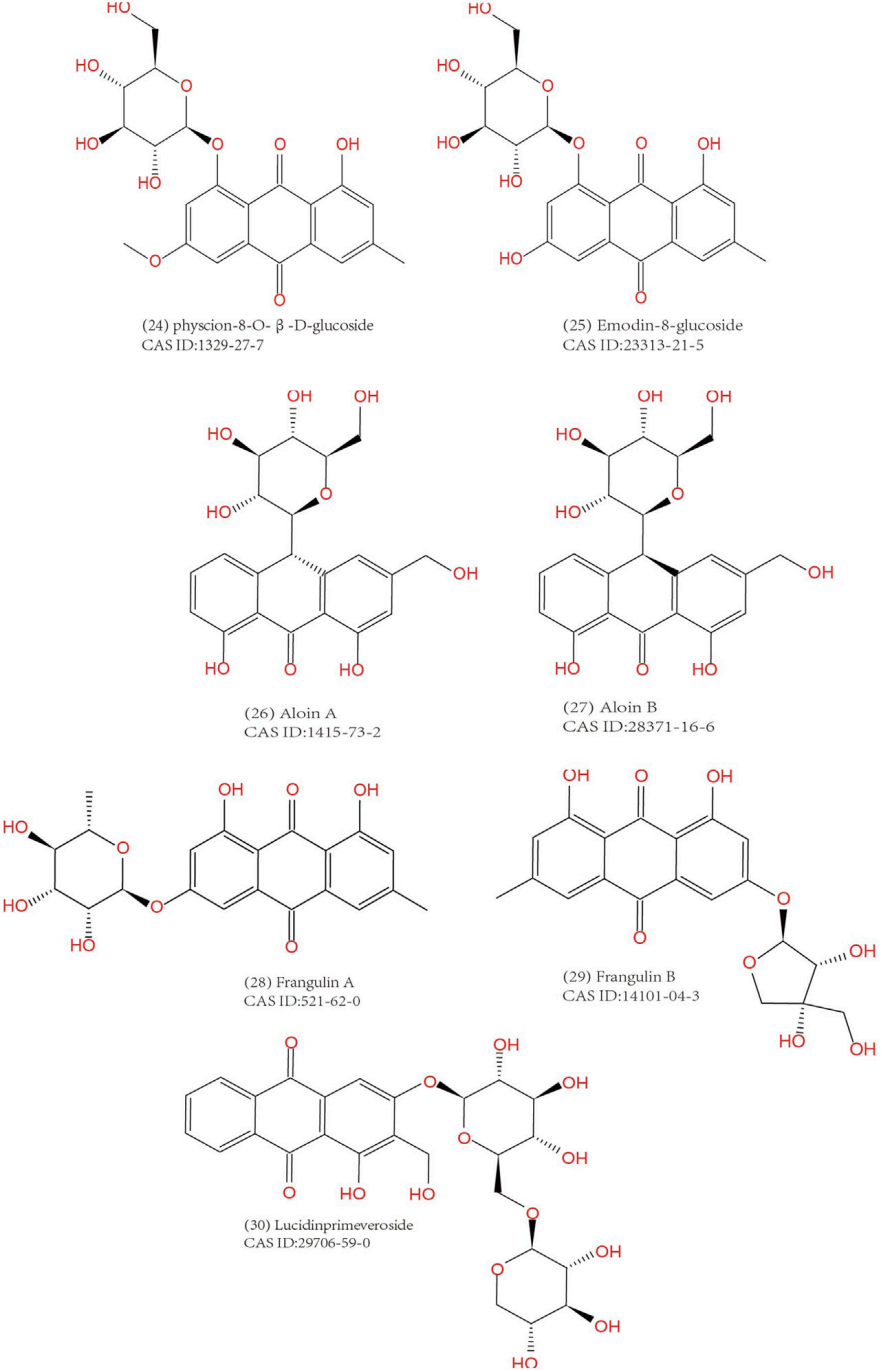
Anthraquinone glucosides extracted from TCM herbs.

**FIGURE 5:**
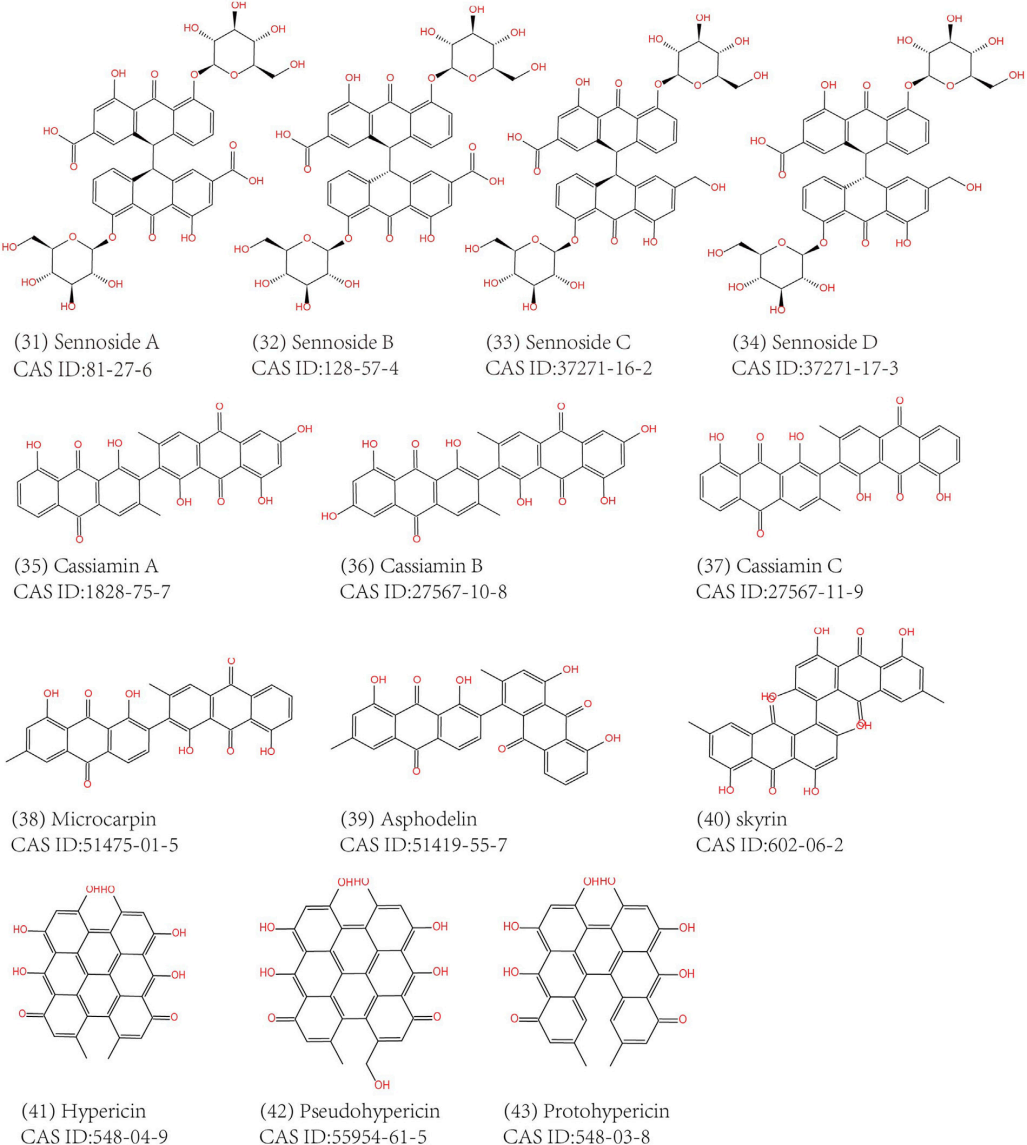
Free anthraquinones containing bi-anthracene nucleus extracted from TCM herbs.

Common anthraquinones isolated from anthraquinone-containing TCM herbs related to MC are shown below (Figures 3–5).

## 3 Traditional Chinese herbs associated with melanosis coli

To get the picture of the general situation of anthraquinone-containing TCM herbs in connection with MC, we performed related information retrieval in CNKI and Pubmed. Our retrieved results showed that there were many MC cases diagnosed by gastrointestinal endoscopy after long-term administration of Chinese herbs like Dahuang, Fanxieye, Shuli (Kew and Chakravarthi, 2013; Bechara et al., 2016; Iwamuro et al., 2020). However, Dahuang and Maidong (Radix Ophiopogonis) are the only two herbs which have been verified to cause MC in animal models. The reason that Shuli (*Rhammus*) and Fanxieye are considered to be associated with MC is that their similar laxative functions and rich contents of anthraquinones. Since the specific mechanism of MC caused by the administration of anthraquinone-containing herbs is still unclear, all the retrieved herbs with isolated anthraquinones or with anthraquinones content determination are summarized in Table 1 to avoid omissions. At the same time, it should not be ignored that long-term or dependent use of anthraquinones might play a critical role in MC pathogenesis, so we made a simple classification of these herbs based on whether they are administrated for a long time or not (Table 1). In the future, it will be necessary to perfect the isolation, identification and determination of anthraquinone compositions from TCM herbs above. We are looking forward to more studies to reveal underlying molecular mechanisms of MC and explore the MC pathogenesis related to these herbs.

**TABLE 1:** Anthraquinone-Containing TCM herbs related to MC.

Family	TCM herbs	Original plants	TCM application	Authenticated constituents of anthraquinones	Quantitative determination of anthraquinones	References
Polygonaceae	Dahuang	Rheum palmatum L., Rheum tanguticum Maxim.ex Balf. and Rheum officinale Baill.	expelling pathogenic heat to loosen the bowels; removing pathogenic heat from the blood and toxic material from the body; dissipating blood stasis and restoring menstruation	emodin, emodin-8-O-β-D-glucopyranoside, aloe-emodin, aloe-emodin glucoside, 6-methyl-aloe-emodin, 6-methyl-aloe-emodin-triacetate, physcion, physcion-8-O-β-D-glucopyranoside, rhein, 6-methyl-rhein, 6-methyl-rhein-diacetate, rhein-8-O-glucoside, chrysophanol, chrysophanol-8-O-β-D-glucopyranoside, revandchinone-3, citreorosein, emodin 8-O-(6′-O-malonyl)-glucoside, emodin 8-O-(2′,3′,4′,6′-tetraacetyl)-glucoside, emodin 8-O-β-D-glucopyranosyl-6-O-sulfate, 2-hydroxymethyl anthraquinone, alizarin	The content of total anthraquinones ranged from 1.11% to 2.08%. (by supercritical fluid chromatography, 2015)	Mohtashami et al. (2021)
						Aichner and Ganzera (2015)
	Heshouwu	Fallopia multiflora (Thunb.) Harald.	relaxing bowels; removing toxicity for eliminating carbuncles; strengthening and nourishing blood and essence	emodin, aloe-emodin, chrysophanol, physcion, rhein, emodin-1,6-dimethyl ether, emodin-8-methyl ether, citreorosein, citreorosein-8-methyl ether, emodin-3-methyl ether fallacinol, emodin-6,8-dimethyl ether, 2-acetylemodin, emodin-8-O-β-D-glucopyranoside, physcion-8-O-β-D-glucopyranoside, emodin-3-methylether-8-O-β-D-glucopyranoside, physcion-8-O-(6′-O-acetyl)-β-D-glucopyranoside, emodin-8-O-(6′-O-acety1)-β-D-glucopyranoside, chrysophanol-8-O-β-D-glucopyranoside	The contents of free and combined anthraquinones respectively ranged from 0.1 mg-g to 1.7 mg-g and from 1.0 mg-g to 8.7 mg-g. (by high performance liquid chromatography, 2021)	(Lin et al., 2015);
						Gao et al. (2021)
	Huzhang	Polygonum cuspidatum Sieb.et Zucc.	dispelling pathogenic wind and moistness from the body; eliminating blood stasis to relieve pain; relieving cough and reducing sputum	anthraglycoside A, anthraglycoside B, chrysophanol-8-O-β-D-glucoside, polyganin A, polyganin B, citreorosein, physcion, emodin, fallacinol, questin, aloe-emodin, questinol, rhein, chrysophanol, xanthorin, isorhodoptilometrin	The average total content of emodin and physcion was 3.79%. (by high performance liquid chromatography, 2017)	Liang et al. (2022);
						Ou et al. (2017)
	Suanmo	Rumex acetosa L., Rumex patientia L., Rumex obtusifolius L. and other similar plants of genus Rumex	removing pathogenic heat from the blood and toxic material from the body; purgation	emodin, chrysophanol, physcion, 1,6,7-trihydroxy-3-methoxy-anthraquinone, 1,3,5-trihydroxy-7-methylanthraquinone, 6-hydroxy-aloe-emodin, chrysophanol-8-O-β-D-glucoside, emodin-8-O-β-D-glucopyranoside	The contents of emodin, aloe-emodin, chrysophanol and physcion respectively ranged from 8.53 mg-g to 54.54mg-g, from 0.17 mg-g to 1.09mg-g, from 1.06 mg-g to 2.60 mg-g and from 0.33 mg-g to 1.34 mg-g. (by ultra-performance liquid chromatography, 2017)	Deng et al. (2016);
						Wang et al. (2017b)
	Hongsanqi	Polygonum suffultum var.pergracile	stopping bleeding by eliminating blood stasis; promoting qi circulation; restoring menstruation	-	The total anthraquinones ranged from 1.0% to 1.5%. (by spectrophotometry, 2015)	Qi et al. (2015)
Fabaceae	Fanxieye	Cassia angustifolia Vahl. and Cassia acutifolia Del.	expelling pathogenic heat to loosen the bowels; purgation; alleviating water retention	sennoside A-D, rhein-1-glucoside, rhein-8-glucoside, rhein, aloe-emodin, aloe-emodin-8-glucoside	The content of total anthraquinones was 1.67%. (by high performance liquid chromatography, 2009)	Oladeji et al. (2020)
						Panichayupakaranant et al. (2009)
	Juemingzi	Cassia obtusifolia L.and Cassia tora L.	clearing away the liver-fire to improve eyesight; relaxing bowels	emodin, chrysophanol, physcion, aloe-emodin, rhein, obtusin, chryso-obtusin, aurantio-obtusin, obtusifolin, questin, alaternin, chrysarobin, 1-desmethylobtusin, 1,2-dihydroxyanthraquinone, 1-hydroxy-3,7-diformylanthraquinone, emodin-6-glucoside, obtusifolin-2-O-β-D-glucoside, aurantio-obtusin-6-O-β-D-glucopyranoside, etc.	The mean contents of obtusifolin-2-glucoside, aurantio-obtusin, obtusifolin, aloe-emodin, rhein, emodin, chrysophanol and physcion were 0.1786mg-g, 1.0146mg-g, 0.2287mg-g, 0.2165mg-g, 0.8132mg-g, 0.2044mg-g, 0.3395 mg-g and 0.0795 mg-g respectively. (by high-performance liquid chromatography-diode array detection, 2011)	Dong et al. (2017)
						Xu et al. (2012)
	Wangjiangnan	Cassia occidentials L.	clearing away the liver-fire; purgation; diuresis; clearing away toxic material from the body to relieve swelling	physcion, emodin, aloe-emodin, etc.	The content of total anthraquinones was 4.069 mg-g. (by ultraviolet spectrophotometry, 2018)	Yadav et al. (2010)
						Guo et al. (2018)
	Huangqi	Astragalus membranaceus (Fisch.) Bge.var.mongholicus (Bge.) Hsiao and Astragalus membranaceus (Fisch.) Bge.	tonifying qi and invigorate vital function; consolidating exterior for arresting sweating; inducing diuresis to alleviate edema; promoting the secretion of saliva or body fluid and nourishing the blood; removing stagnation to dredge blockage of meridians; evacuating pus and expelling toxin by reinforcing qi and blood; regenerating tissue to heal wond	chrysophanol, emodin, physcion, aloe-emodin	The content of total anthraquinones was 0.5047%. (by differential spectrophotometry, 2017)	Jiang (2017)
						Zhao et al. (2003)
Liliaceae	Luhui	Aloe barbadensis Miller., Aloe ferox miller and other closely related plants of genus Aloe.	purgaiton; clearing liver-fire	aloin A, aloin B, emodin, aloe-emodin	The content of total anthraquinones was 13.837 mg-g. (by ultraviolet spectrophotometry, 2012)	Gao et al. (2019)
						Wang et al. (2012)
	Maidong	Ophiopogon japonicus (L.f) KerGawl.	nourishing yin and promoting the secretion of saliva or body fluid; clearing away the heart-fire; moiste ning the lung	physcion, emodin	-	Cheng et al. (2005)
	Huangjing	Polygonatum kingianum Coll.et Hemsl., Polygonatum sibiricum Red.and Polygonatum cyrtonema Hua.	tonifying qi and nourishing yin; strengthening the spleen; moistening the lung; tonifying the kidney	-	The mean contents of free anthraquinones and combined anthraquinones were respectively 0.53% and 0.0054%. (by spectrophotometry, 2012)	Peng et al. (2012)
Rhamnaceae	Shuli	Rhamnus dahurica Pall., Rhamnus ussuriensis J.Vass.and other plants of genus Rhamnus.	purgation; clearing away heat and removing moistness through promoting diuresis	physcion, chrysophanol, emodin, rhein, aloe-emodin, alaternin, cynodontin, frangulin A, frangulin B, glucofrangulin A, glucofrangulin B, anthraquinone glycosides	The contents of emodin, aloe-emodin, chrysophanol, rhein and physcion were respectively 1.163mg-g, 0.041mg-g, 0.115mg-g, 0.553 mg-g and 0.546 mg-g. (by high performance liquid chromatography, 2010)	Nekkaa et al. (2021)
Rubiaceae	Qiancao	Rubia cordifolia L.	removing pathogenic heat from the blood; removing blood stasis; hemostasis; restoring menstruation	alizarin, lucidinprimeveroside, munjistin, nordamnacanthal, physcion, pupurin, rubiadin, soranjidiol, tectoquinone, xanthopurpurin, rubiacordone A, 1-hydroxy-2-methylanthraquinone, 1,4-hihydroxy-2,3-dimethylanthraquinone, 1,3,6-trihydroxy-2-methylanthraquinone-3-O-α-rhamnosyl-(1→2)-β-glucoside, etc.	The content of anthraquinones ranged from 3.37% to 3.73%. (by differential spectrophotometry, 2011)	Shan et al. (2016)
						Wei et al. (2011)
	Bajitian	Morinda officinalis How.	reinforcing the kidney-yang; strengthening the bones and muscles; eliminating pathogenic wind and damp	physcion, rubiadin, tectoquinone, digiferruginol, lucidin-ѡ-ethyl ether, 1-hydroxy-2-methyl-anthraquinone, 1-hydroxy-2,3-dimethyl-anthraquinone, alizarin-2-methyl ether, etc.	The contents of total anthraquinones was 1.158 mg-g. (by spectrophotometry, 2014)	Jing et al. (2014)
	Baihuasheshecao	Oldenlandia diffusa (willd.) Boxb.	clearing away heat and toxic materials; detumescence; diuresis	2,3-dimethoxy-6-methylanthraquinone, 2,7-dihydroxy-3-methyl anthraquinone, 1,3-dihydroxy-2-methylanthraquinone, Robustaquinone D (1,7-dihydroxy-6-methoxy-2-methylanthraquinone)	The contents of 2-methy-3-methoxyanthraquinone and 2,3-dimethoxy-6-methyanthraquinone ranged from 0.26 mg-g to 0.85 mg-g and from 0.25 mg-g to 0.68 mg-g. (by high-performance liquid chromatography-diode array detection, 2014)	Li (2016)
						Cao et al. (2014)
Solanaceae	Digupi	Lycium chinense Mill. and Lycium barbarum L.	clearing away pathogenic heat from the body to expelhectic fever; cleari ng away the lung-heat and purging fire	emodin, physcion, 6-hydroxyrubiadin, 3-O-(2-O-α-l-rhamnopyranosyl-6-O-acetyl-β-d-glucopyranosyl)-6-hydroxy-rubiadin	The content of total anthraquinones ranged 1.15%–5.73%. (by colorimetry, 2008)	Qian et al. (2017)
						Ma et al. (2008)
Lardizabalaceae	Daxueteng	Sargentodoxa cuneata (oliv.)Rehd.et wils.	clearing away heat and toxic materials; promo ting blood circul ation; alleviat ing pain by expelling pathogenic wind	physcion, emodin, chrysophanol	The content of total anthraquinones was 0.00284%. (by colorimetry, 2019)	Zhang et al. (2021a)
						Wei et al. (2019)

We are inclined to think that not only TCM herbs containing anthraquinones but also long-term oral administration is an etiological factor. Below, we provide a detailed introduction of herbs that meet the above conditions. Table 1 shows a partial list of TCM herbs containing anthraquinones. We review their use and application in TCM. In this section, we described the herbs of the same genera together, for the reason that the herbs of same genera may contain anthaquinones with similar classes and contains, and this approach also systematically convey the information of original plants of Chinese herbs to readers lacking knowledge of TCM. Moreover, A short list of Chinese patent medicines is presented Table 2.

**TABLE 2:** Chinese patent medicine containing anthraquiones.

Chinese patent medicine	Indications	Anthraquinone-containing Components
San Huang tablets	upper respiratory tract infection, constipation, oral ulcer	*Rhei radix et rhizoma*
Da Cheng Qi decoction	acute simple intestinal obstruction, acute cholecystitis, acute respiratory distress syndrome, acute appendicitis	*Rhei radix et rhizoma*
Da Huang Mu Dan decoction	acute simple appendicitis, acute biliary tract infection, biliary ascariasis, pancreatitis, acute pelvic inflammation	*Rhei radix et rhizoma*
Dang Gui Long Hui pills	constipation, essential hypertension, hepatitis, oral ulcer	*Rhei radix et rhizoma, Aloe*
Ma Ren Run Chang pills	constipation	*Rhei radix et rhizoma*
Niu Huang Jie Du pellets	upper respiratory tract infection, buccal inflammation	*Rhei radix et rhizoma*
Jiu Zhi Da Huang pills	urinary infection, constipation	*Rhei radix et rhizoma*
Dao Chi pills	urinary infection, furuncle,carbuncle	*Rhei radix et rhizoma*
Da Huang Zhe Chong pills	hepatitis, liver cirrhosis, liver cancer, advanced abdominal/pelvic solid tumors	*Rhei radix et rhizoma*
Tong Bian Ling capsules	constipation	*Folium Sennae*
Composite Aloe capsules	constipation, anxiety	*Aloe*
Shuang Hu Qing Gan granules	constipation, hepatitis	*Rhizoma polygoni cuspidati, Oldenlandia diffusa*
Dan Ning tablets	chronic cholecystitis, constipation	*Rhei radix et rhizoma, Rhizoma polygoni cuspidati*
Wei Xue Ning mixture	thrombocytopenia, leukopenia,anemia	*Spatholobus suberectus, Rhizoma polygoni cuspidati*
Cong Rong Tong Bian oral liquid	constipation	*Polygonum multiflorum*
Qi Bao Mei Ran pellets	alopecia, male Infertility	*Polygonum multiflorum*
Geng Nian An tablets	climacteric syndrome	*Radix Polygoni Multiflori, Caulis Polygoni Multiflori, Radix Ophiopogonis*
Shi Yi Wei Shen Qi tablets	chronic cholecystitis, dyspepsia,fatigue	*Radix Astragali, semen cassiae*

### 3.1 Polygonaceae

#### 3.1.1 Rhei Radix et Rhizoma (Dahuang)

Rhei Radix et Rhizoma, also called Dahuang in China, derives from the roots and rhizome of Rheum palmatum L., Rheum tanguticum Maxim. ex Balf., and Rheum officinale Baill. Dahuang has been used in the TCM field for thousands of years to treat coagulated heat by purgation and clearing heat-fire. The potency of Dahuang is distinguished by the different processing method. The toxicity and side effects of Cooked Dahuang and Dahuang Parched in Wine are less than Dahuang.

The main anthraquinone chemical components of *Dahuang* include rhein (3), chrysophanol (2), emodin (1), aloe-emodin (5), physcin (4), and sennoside A–D (31–34)). Aichner and Ganzera determined that the free anthraquinone content in Dahuang ranged from 0.32% to 0.73%, and the anthraquinone content after hydrolysis in Dahuang extract rose to 2.1% (Aichner and Ganzera, 2015). The content of combined anthraquinones in Dahuang will be reduced after being processed at a high temperature because of the poor water solubility of free anthraquinones in this condition. Dahuang is the main composition of several commonly used Chinese medicinal prescriptions, such as the Da Cheng Qi decoction and its derivative decoction, the Da Huang Mu Dan decoction and the Da Huang Fu Zi Xi Xin decoction. Dahuang is commonly used as a laxative herb in both traditional and modern medicine. Some Chinese patent medicines present on the market, like San Huang tablets, Dang Gui Long Hui pills, and Ma Ren Run Chang pills, have been abused for the treatment of constipation.

It was demonstrated that low-dose (1 g/kg) and high-dose (4 g/kg) extract of Dahuang cause inflammatory changes such as neutrephil infiltration, and exfoliation of mucosal epithelial cells in colonic tissue (Zhang, 2013). Mitochondrial swelling, lymphocyte/leukocyte invasion induced by low-dose extract of Dahuang could be observed under the electron microscope (Zhang, 2013). High-dose extract of Dahuang significantly increased the expression of Bax and the apoptosis index of colon of guinea pigs (Zhang, 2013). Another study showed that extract of Dahuang with a dosage of 3–24 g/kg for 60 days induced MC in a dose-dependent fashion in guinea pigs, and the degree of induced MC was positively correlated with the apoptosis rate and TNF-α level in colonic epithelium (Chen and Gang, 2011).

#### 3.1.2 Radix Polygoni Multiflori (Heshouwu) and Caulis Polygoni Multiflori (Yejiaoteng)

Heshouwu and Yejiaoteng come from the same plant called Fallopia multiflora (Thunb.) Harald. or Polygonum multiflorum Thunb., a perennial twining vine of the polygonum family. Heshouwu refers to the underground portion, and Yejiaoteng refers to the aboveground portions.

The predominant anthraquinones in Heshouwu are emodin-type anthraquinones, such as emodin, aloe-emodin, chrysophanol, physcion rhein, 1,6-dimethyl ether-emodin,emodin-8-methyl ether (14), citreorosein (13), citreorosein-8-methyl ether (Lin et al., 2015). Gao et al. (Gao et al., 2021) measured the content of anthraquinones in Heshouwu from various producing areas. The authors concluded that the contents of free and combined anthraquinones ranged from 0.1 mg/g to 1.7 mg/g and 1.0 mg/g to 8.7 mg/g, respectively. Moreover, they found that processing increased the emodin content in Heshouwu, but the reasons remain uncertain.

Heshouwu is a traditional Chinese herb with potency to nourish the liver and kidney, replenish essence and blood, blacken the beard and hair, strengthen muscles and bones, detoxify and relax the bowels. It is a common herb present in most tonic Chinese patent medicines, mainly available as over-the-counter (OTC) preparations in China, such as Qi Bao Mei Ran pellets, Geng Nian An tablets, Shi Yi Wei Shen Qi tablets, and Ren Shen Zai Zao pills. There are also some preparations with the effects of strengthening the body containing Heshowu Although Yejiaoteng and Heshouwu are from the same source, their Chinese medical pharmacology differs slightly from each other. Yejiaoteng belongs to wind-damp-dispelling medicine, and due to its tonic effect, it is often used to treat wind syndrome caused by blood deficiency. These two herbs are also used to relieve constipation caused by the deficiency of Yin and blood in traditional Chinese theories.

#### 3.1.3 Rhizoma Polygoni Cuspidati (Huzhang)

In China, Rhizoma Polygoni Cuspidati, called Huzhang, is obtained from dried roots and the rhizome of Polygonum cuspidatum Sieb. et Zucc. Huzhang effectively relieves jaundice by expelling moistness and removing pathogenic heat and toxicity. It is often prescribed externally for bruises, bruising, swelling, and pain, and orally for jaundice, hepatitis, and cholelithiasis in the Li Dan Pai Du decoction and the Bie Jia decoction. It can also be used to treat constipation with dry stools. The content of anthraquinones in Huzhang by HPLC (High Performance Liquid Chromatography) is approximately 3.79%.

#### 3.1.4 Rumex (Suanmo)

Called Suanmo in China, plants of the Rumex genus are used not only in TCM but also as a vegetable with the functions of removing pathogenic heat and toxic materials. Due to its purgative effect, it is commonly used to relieve constipation. Wang et al. (Wang J. F. et al., 2017) measured the contents of several kinds of anthraquinones in the Suanmo by Ultra Performance Liquid Chromatography. Their results showed that the contents of emodin, aloe-emodin, chrysophanol, and physcion were 8.53 mg/g to 54.54 mg/g, 0.17mg to 1.09 mg/g, 1.06 mg/g to 2.60 mg/g, and 0.33 mg/g to 1.34 mg/g, respectively. As a widely used herb, the side effects caused by its anthraquinones constituents also deserve greater attention.

#### 3.1.5 Radix Polygonum Suffultum (Hongsanqi)

Hongsanqi, from the dried rhizome of Polygonum suffultum var. Hongsanqi has been used to stop bleeding, regulate menstruation, and relieve traumatic swelling and pain in TCM. The constituents content measurement in a pharmacological study in Hongsanqi showed that the total anthraquinones content was 1.0%–1.5% (Qi et al., 2015). When used for treating trauma, it is usually ground into a powder and then soaked in wine and taken as an oral medicine. In this preparation, the extraction ratio of anthraquinones will be higher than that in water solution because alcohol is an organic solvent.

### 3.2 Fabaceae

#### 3.2.1 Folium Sennae (Fanxieye)

Folium Sennae, called Fanxieye in China, with the familiar name Senna, are dry leaflets of Cassia angustifolia Vahl. and Cassia acutifolia Del. Fanxieye is one of the most commonly used herbs for constipation. In recent years, driven by economic benefit, this herb has been marketed and touted as a remedy for weight loss, detoxification, and beauty for those who want to lose weight and improve their appearance. In addition, it even appears as a kind of flavor vegetable in some middle Eastern and Mediterranean countries. There is no such abuse of Fanxieye in China, and Fanxieye is prescribed more frequently by professional traditional Chinese physicians.

The chemical compositions in the extracts of Fanxieye are mainly anthraquinones, such as sennoside A–D, rhein, aloe-emodin, and chrysophanol. Panichayupakaranant et al. reported that the content of anthraquinones in Fanxieye is 1.67% by HPLC (Panichayupakaranant et al., 2009). Abuse of Fanxieye can not relieve constipation but may exacerbate abdominal pain, bloating, and irregular bowel movements and form a vicious circle.

A prospective study showed that child patients taking sennosides to treat functional constipation had a higher morbidity of MC, with an adjusted odds ratio 13.88 (Chen et al., 2022). It was reported that intragastric administration of 100 mg/kg per day sennoside for 12 weeks induced lipofuscin deposition and colonic melanosis (Shen, 2017). Long-term administration of Sennoside A facilitates apoptosis and inflammation of colon epithelial cells through increasing the mRNA level of IL-1β,IL-6,IL-10, and TNF-α, and decreasing the expression of Ki67(Shen, 2017). It is worth noting that the effect of Sennoside A is dependent on the concentration of intestinal butyric acid and the abundance of intestinal butyricogenic bacteria (Shen, 2017). van Gorkom et al. demonstrated that Sennosides induced colonic epithelial cell apoptosis through p53 and p21/WAF signaling pathway, which may be a potential target for suppressing apoptosis in MC (van Gorkom et al., 2001).

#### 3.2.2 Semen Cassiae and Cassia mimosoides (Juemingzi or Shanbiandou)

Semen Cassiae, with the Chinese name “Juemingzi,” is from the mature seeds of Cassia obtusifolia L. or Cassia tora L. Shanbiandou are dry aerial parts of Cassia mimosoides L. and are also obtained from the genus Cassiae.

Recent studies have shown that its purgative effect is due to its abundant anthraquinones as a stimulant and oil lubricant. Xu et al. discovered the content of seven free anthraquinones and one combined anthraquinones in Juemingzi by HPLC-diode array detection (HPLC-DAD) (Xu et al., 2012). Their results showed that the total contents of eight anthraquinones were 3.075 mg/g.

Juemingzi has been used in East Asian countries (China, Japan, Korea.) for thousands of years to ‘clear liver fire’, which means relieving some symptoms of cardiovascular and cerebrovascular diseases and improving complications caused by hyperglycemia and hyperlipidemia. Moreover, with research on its pharmacology increasingly perfected, Juemingzi has become the favorite choice as a healthy tea beverage in recent years. Many people consistently ignore the side effects of chronic administration of Juemingzi extracts. This risk factor is exacerbated by a lack of medical knowledge or the deliberate concealment of the side effects of Juemingzi by many ‘Jue Ming Zi tea’ beverage producers. In recent years, MC caused by the long-term administration of Juemingzi has become more and more common. However, Shanbiandou is not so much abused, which is used mainly as a diuretic drug to alleviate water retention in patients with nephritis edema.

Wangjiangnan, mature seeds of Cassia Occidentials L., with similar appearance and TCM effects to Juemingzi, is used as a substitute for Juemingzi to relieve constipation. However, more frequent and significant side effects and toxicity have been reported, for which its application is limited. Modern pharmacological studies have revealed that emodin, aloe-emodin, and physcion are the main effective constituents of Wangjiangnan (Yadav et al., 2010). Quantitative determination of total anthraquinones by ultraviolet spectrophotometry in Wangjiangnan is 4.096 mg/g (Guo et al., 2018).

#### 3.2.3 Radix Astragali (Huangqi)

*Radix Astragali*, called *Huangqi* in China, is made from dried roots of Astragalus membranaceus (Fisch.) Bge. *var*. *mongholicus* (Bge.) Hsiao and Astragalus membranaceus (Fisch.) Bge. Many researchers have not linked *Huangqi* with anthraquinones because *Huangqi* is a well-known qi-invigorating herb with the power to boost immunity, and the capacity to protect liver and kidney. Due to its potency to tonify the body and strengthen health, Huangqi is usually present in many daily healthcare products. There have been reported to be several types of anthraquinones with a concentration of 0.5047% in Huangqi (Zhao et al., 2003; Jiang, 2017).

### 3.3 Liliaceae

#### 3.3.1 Aloe (Luhui)

Aloe, called Luhui in China, native to South Africa and South America, is derived from the leaves of Aloe barbadensis Miller., Aloe ferox Miller, and other closely related plants of the genus Aloe. In TCM, Luhui refers to the dried and concentrated sap of Aloe leaves. Luhui is mainly used to promote defecation and clear liver fire. The sap of Luhui is also an externally applied agent for anaphylaxis, microbial infections, and inflammation of local skin and promotes wound healing and skincare.

Anthraquinones are the primary composition in the extract of Luhui, including aloin A (26), aloin B (27), aloe-emodin, emodin, aloin, and Aloesaponol I, II, III, IV (Gao et al., 2019). Wang et al. reported that the content of total anthraquinones in Luhui was 13.837 mg/g. Moreover, the content of combined anthraquinones in different prepared Luhui are directly drying, stir-frying, stir-frying with wine, stir-frying with salt, stir-frying with vinegar, and steaming with vinegar from the highest to the lowest (Wang et al., 2012).

Luhui extract often appears on the ingredient lists of many cosmetics, skincare, and beauty products. Some companies add Luhui to dairy products, tea-based beverages, and desserts to provide detoxifying, beautifying, and weight loss functions. Some combine Luhui, Fanxieye and Juemingzi to make teabags possessing the effects of excreting fat. Furthermore, these applications expose individuals to higher risk of Laxative Abuse Syndrome and MC. Therefore, Luhui is not a suitable or completely safe herb for chronic oral administration. Case reports of MC with the habit of oral administration of Aloe are not uncommon (Iwamuro et al., 2020).

#### 3.3.2 Rhizoma Polygonati (Huangjing)

Huangjing, or Rhizoma Polygonati, is a Yin-tonifying herb obtained from dried roots and the rhizome of Polygonatum kingianum Coll. et Hemsl., Polygonatum sibiricum Red. and Polygonatum cyrtonema Hua. With the effects of stimulating qi and nourishing yin, strengthening the spleen, moistening the lung and tonifying the kidney, Huangjing is a good remedy choice in TCM for fatigue, hemoptysis tussiculation, and weakness of the waist and knees. Peng et al. showed that the average contents of free anthraquinones and combined anthraquinones of Huangjing are 0.53% and 0.0054%, respectively (Peng et al., 2012).

#### 3.3.3 Radix Ophiopogonis (Maidong)

Radix Ophiopogonis (Maidong) is prescribed in the Chinese medical diet for clearing heat and arresting coughing. The extract of Maidong with a dosage of 1.5 g/kg∼6 g/kg led to colonic mucosal inflammation and excessive apoptosis in guinea pigs (Zhang, 2013). Although the concentration of emodin in Maidong is less than Dahuang, the extract of Maidong with a dosage of 1.5 g/kg was also able to induce a marked increase in the expression of Bcl-2 in colonic epithelium in guinea pigs (Zhang, 2013).

### 3.4 Rhamnaceae

Shuli is derived from mature fruits, leaves, or bark of Rhamnus dahurica Pall., Rhamnus ussuriensis J. Vass. and other plants of the genus Rhamnus. In the theory of TCM, it is used to remove intestinal heat and activate detoxicification. Another use of Shuli is to relieve constipation, which is the most common application of Shuli in traditional medicine from other countries, except in China. Rich in anthraquinones with a classical structure, Shuli is an irritant laxative (Nekkaa et al., 2021). MC patients with chronic use of Rhamnus bark and leaves have been widely reported. MC of patients with medication history of Shuli are mainly detected by routine digestive endoscopy (Bechara et al., 2016). Interestingly, as long as these laxatives are abandoned, MC will spontaneous relive to normal appearance (Kew and Chakravarthi, 2013).

### 3.5 Rubiaceae

#### 3.5.1 Rubiae Radix et rhizoma (Qiancao)

Rubiae Radix et Rhizoma, called Qiancao in China, is from the dried roots and rhizome of Rubia cordifolia L. As a hemostatic herbs, Qiancao is used to treat hemorrhage caused by blood heat with blood stasis in TCM. After being stir-fried, its drug property will be milder and has become one of the most effective herbs for regulating menstruation and stopping bleeding for female patients. Oil and wine containing Qiancao for medical use are effective external agents for pain in joints and bones caused by blood stasis. Wei et al. determined that the content of total anthraquinones in Qiancao ranged from 3.37% to 3.73% (Wei et al., 2011). Zheng et al. treated normal rats with a sequential dosage of 70% ethanol extract of Qiancao (5/10/30 g/kg) for 60 days, which was respectively equivalent to 6/12/36 times the prescribed dosage of human. Their results showed that even though the dosage of Qiancao had reached 30 g/kg, it still could not cause MC in rats (Zheng, 2017). More dose-ranging studies are needed to clarify whether Qiancao can cause MC. Even so, Patients who use Chinese patent medicines and herbal prescriptions containing Qiancao to treat hemorrhagic disorders should be mindful, especially for those with long medication cycles to treat gynecological diseases.

Xiaohongshen, from Rubia yunnanensis Diels, whose usage is similar to Qiancao, can replace Qiancao in prescriptions. 1,3,6-trihydroxy-2-methyl anthraquinone, munjistin (18), lucidin (11), 1-hydroxy-2-methyl anthraquinone, 1,3-dihydroxy-2-methyl anthraquinone, xanthopurpurin (19) and 1,4-dihydroxy-2-hydroxymethylanthraquinone have been identfied in its preparations (Wang, 2012).

#### 3.5.2 Radix Morindae Officinalis (Bajitian)

Bajitian or Radix Morindae Officinalis, a Yang-tonifying herb, is the dried root of Morinda officinalis How. Bajitian possesses the TCM effects of recharging the kidney, strengthening bones and muscles, and dispelling wind and moistness. As an adjuvant therapy, the prescriptions containing Bajitian is helpful in the treatment of impotence, erectile dysfunction, male infertility, and oligospermia. The content of anthraquinones in Bajitian was reported to be 1.518 mg/g. Processing technologies can reduce the content of combined anthraquinones and increase the content of free anthraquinones (Jing et al., 2014). From the standpoint of preventing MC, processed Bajitian is safer than crude Bajitian.

Morinda umbellata (Yangjiaoteng), as a succedaneum of Bajitian, can effectively alleviate symptoms of rheumatic diseases, such as rheumatoid arthritis, gouty arthritis, and rheumatic low back pain. Li et al. extracted 1,6-dihydroxy-2-methoxymethylanthraquinone, and several other anthraquinones with similar chemical structures from Yangjiaoteng (Li et al., 2019).

#### 3.5.3 Oldenlandia diffusa (Baihuasheshecao)

The entire dried plant of Oldenlandia diffusa (willd.) Boxb. is called baihuasheshecao in China. It is a detoxifying herb applied in the traditional medical treatment for insect bites, snake bites, and various inflammatory diseases. Its anti-tumor effects has been discovered in recent years, so its application in the neoplastic diseases is becoming more common. Several anthraquinones, such as 2,3-dimethoxy-6-methyl anthraquinone and 2,7-dihydroxy-3-methyl anthraquinone, have been identified in this herb (Li, 2016). The contents of 2-methyl-3-methoxy anthraquinone and 2,3-dimethoxy-6-methyl anthraquinone range from 0.26 mg/g to 0.85 mg/g and from 0.25 mg/g to 0.68 mg/g, respectively (Cao et al., 2014).

### 3.6 Solanaceae

Digupi, or Cortex Lycii, is the dried root bark of Lycium chinense Mill. or Lycium barbarum L. Digupi has a dual effect on heat removal and Yin tonification, so in TCM, it is used to treat fever, night sweats, diabetes, heart disease, gynecological diseases, and neurasthenia. Ma et al. determined that the content of total anthraquinones in Digupi ranged from 1.15% to 5.73% (Ma et al., 2008). In addition, the older the cultivation of Digupi is, the higher content of anthraquinones Digupi contains. As a key ingredient, Digupi is present in some Chinese patent medicines with the effects of resisting fatigue and aging, strengthening tendons and bones, and improving reproductive function. These drugs are likely to be taken over the long-term by middle-aged and elder patients.

### 3.7 Lardizabalaceae

Daxueteng, or Caulis Sargentodoxae, is derived from vines and stems of Sargentodoxa cuneata (oliv.) Rehd. et Wils., which is widely distributed in the subtropical region of China. As a heat-clearing drug in TCM, it is empirically applied in treating amenorrhea caused by blood-heat, infectious skin diseases, appendicitis, and wind-heat type rheumatic arthralgia. Isolated anthraquinone were mainly divided into emodin, chrysophanol and physcion (Zhang W. et al., 2021). The total anthraquinones content in Daxueteng measured by colorimetry was 0.00284% (Wei et al., 2019).

## 4 Conclusion and future perspectives

This review summarizes risk factors and etiologies of MC and some traditional Chinese herbs that may be associated with the onset and development of MC. The most acceptable views accounting for MC development are anthraquinones abuse and constipation. The pathogenesis is mainly attributed to increased colonic epithelial apoptosis. Currently, studies supporting the apoptosis hypothesis are more advanced than others, but the underlying molecular mechanisms corresponding to each herb still require further studies. This review provides an overview of anthraquinones-contained Chinese herbs that cause MC. Dahuang (Rhei Radix et Rhizome), Heshouwu (Radix Polygoni Multiflori), Huzhang (Rhizoma Polygoni Cuspidati), Juemingzi (Semen Cassiae), Luhui (*Aloe*)*,* and Qiancao (Rubiae Radix et Rhizoma) are herbs with the most abundant anthraquinones known to date. Unfortunately, the isolation, identification and content determination of anthraquinones for most TCM herbs need to be perfected. Another limitation is that the specific molecular mechanism by which each herb cause MC is still an undeveloped territory. Our advice on using these herbs with caution is mainly based on the fact that they contain anthraquinones and are commonly used in TCM. Traditional Chinese physicians should avoid heavily using those herbs or Chinese patent drugs to alleviate chronic symptoms for too long. The daily dose of short-term application of anthraquinones in adults to relieve constipation should be restrained within 30 mg/d and 2–3 times per week (Younes et al., 2018), and any use time of anthraquinones laxatives longer than 1–2 weeks requires medical supervision. Under the condition of related producers have given a clear indication of anthraquinones contents and suggestive doses, users can regulate their daily doses of administration according to this information. Lifestyle modification, dietary fiber supplementation, pelvic muscle exercise, and fecal microbiota transplantation are optional and harmless non-pharmaceutical therapies for constipation. It is necessary to strengthen education on the rational use of natural laxatives among medical workers. Anthraquinone-containing TCM herbs, prescription drugs, food supplement and healthcare products need a more strict production and application management. Finally, further studies on toxicity-structure relationship between anthraquinone compounds and MC need to be carried out to further elucidate the pathogenesis of MC.

## References

[B1] Abu Baker F. Mari A. Feldman D. Suki M. Gal O. Kopelman Y. (2018). Melanosis coli: A helpful contrast effect or a harmful pigmentation? Clin. Med. Insights Gastroenterol. 11, 1179552218817321. 10.1177/1179552218817321 30574001 PMC6299301

[B2] Aichner D. Ganzera M. (2015). Analysis of anthraquinones in rhubarb (Rheum palmatum and Rheum officinale) by supercritical fluid chromatography. Talanta 144, 1239–1244. 10.1016/j.talanta.2015.08.011 26452953

[B3] Balázs M. (1986). Melanosis coli. Ultrastructural study of 45 patients. Dis. Colon Rectum 29 (12), 839–844. 10.1007/bf02555359 3792165

[B4] Bechara R. Marcon N. Streutker C. J. (2016). Melanosis coli: A disappearing act. Gastrointest. Endosc. 83 (6), 1296–1298. 10.1016/j.gie.2015.10.050 26555299

[B5] Byers R. J. Marsh P. Parkinson D. Haboubi N. Y. (1997). Melanosis coli is associated with an increase in colonic epithelial apoptosis and not with laxative use. Histopathology 30 (2), 160–164. 10.1046/j.1365-2559.1997.d01-574.x 9067741

[B6] Cao G. S. Yang P. M. Zhang J. Y. Li F. Ni J. Gao P. (2014). Determination of two anthraquinones in hedyotidis herba by HPLC-DAD. Chin. J. Exp. Tradit. Med. Formulae 20 (20), 54–56. 10.13422/j.cnki.syfjx.2014200054

[B7] Cha J. M. Lee J. I. Joo K. R. Jung S. W. Shin H. P. (2009). Melanosis ilei associated with chronic ingestion of oral iron. Gut Liver 3 (4), 315–317. 10.5009/gnl.2009.3.4.315 20431767 PMC2852718

[B9] Chen J. J. Kitzia Colliard R. N. Nurko S. Rodriguez L. (2022). Melanosis coli is not associated with colonic dysmotility nor severity of pediatric functional constipation. Dig. Dis. Sci. 67 (8), 3922–3928. 10.1007/s10620-021-07191-z 34379221

[B10] Chen J. Y. Pan F. Zhang T. (2009). Rhubarb induced change of tumor necrosis factor-alpha level in Guinea pig model of melanosis coli and its significance. Chin. J. Integr. Tradit. West. Med. Chin. Ed.) 29 (2), 140–142. 19382475

[B11] Chen J. Y. Pan F. Zhang T. Xia J. Li Y. J. (2011). Experimental study on the molecular mechanism of anthraquinone cathartics in inducing melanosis coli. Chin. J. Integr. Med. 17 (7), 525–530. 10.1007/s11655-011-0786-z 21725878

[B12] Chen Z. F. Gang A. S. (2011). Content of active component in wild medicinal plant polygonum ciliinerve determined by HPLC and their relationship with tuber size. J. Hebei Agric. Sci. 15 (01), 165–167. 10.16318/j.cnki.hbnykx.2011.01.059

[B13] Cheng Z. H. Wu T. Li L. Z. Liu N. Yu B. Y. Xu L. S. (2005). in Studies on the liposoluble components from tuber of Ophiopogon japonicus Chin. Pharm. J. (Beijing, China), 20–24.

[B14] Deng Y. H. Sun Y. N. Guo D. D. Ni Y. (2016). Advances in active ingredients and indicative components of Chinese herbs from Rumex. Chin. J. Ethnomed. Ethnopharm. 25 (17), 41–44.

[B16] Dong X. Fu J. Yin X. Yang C. Zhang X. Wang W. (2017). Cassiae semen: A review of its phytochemistry and pharmacology (review). Mol. Med. Rep. 16 (3), 2331–2346. 10.3892/mmr.2017.6880 28677746 PMC5547955

[B17] Dore M. P. Villanacci V. Manca A. Soro S. Schiavo-Lena M. Sabatino G. (2014). Cherry-tree colon: Colonoscopic appearance suggesting drug-induced mucosal injury. Intern. Emerg. Med. 9 (4), 405–409. 10.1007/s11739-013-0930-1 23494541

[B18] Gao H. Y. Yang J. B. Sun H. Song Y. F. Cheng X. L. Wang X. T. (2021). Determination of anthraquinones in Polygoni Multiflori Radix from different origins and processed differently by HPLC. Chin. J. Pharmacovigil., 1–11.

[B19] Gao Y. Kuok K. I. Jin Y. Wang R. (2019). Biomedical applications of Aloe vera. Crit. Rev. Food Sci. Nutr. 59 (1), S244–s256. 10.1080/10408398.2018.1496320 29999415

[B20] Grilo I. Torres-Gómez J. Gómez-Regife L. (2014). Atypical melanosis coli resembling the appearance of cheetah skin. Endoscopy 46 (1), E437–E438. UCTN. 10.1055/s-0034-1377427 25314182

[B21] Guo Y. Li J. Xu J. Zhuang T. (2018). Optimization of extraction process of anthraquinone from Cassia occidentalis L. And DPPH free radical scavenging experiment. Chin. Arch. Tradit. Chin. Med. 36 (02), 428–431. 10.13193/j.issn.1673-7717.2018.02.045

[B22] Hoshi O. Iwanaga T. Fujino M. A. (1996). Selective uptake of intraluminal dextran sulfate sodium and senna by macrophages in the cecal mucosa of the Guinea pig. J. Gastroenterol. 31 (2), 189–198. 10.1007/bf02389517 8680538

[B23] Iseki K. Ishikawa H. Suzuki T. Murakami T. Otani T. Ishiguro S. (1998). Melanosis coli associated with ingestion of bamboo leaf extract. Gastrointest. Endosc. 47 (3), 305–307. 10.1016/s0016-5107(98)70333-5 9540889

[B24] Iwamuro M. Tanaka T. Okada H. (2020). Melanosis coli due to aloe vera consumption. Intern Med. 59 (20), 2633–2634. 10.2169/internalmedicine.5183-20 32641661 PMC7662051

[B25] Jiang P. (2017). Determination of total anthraquinone content in Radix Astragalus. Heilongjiang Med. J. 30 (02), 243–246. 10.14035/j.cnki.hljyy.2017.02.003

[B27] Jing H. Y. Cui N. Shi J. Jia T. Z. (2014). Comparation in content of anthraquinones in Morinda officinalis and other different processing products. Asia-Pac. Tradit. Med. 10 (01), 21–23.

[B29] Johnson J. E. Carpenter B. L. Benton J. Cross R. Eaton L. A. Jr. Rhoads J. M. (1991). Hemorrhagic colitis and pseudomelanosis coli in ipecac ingestion by proxy. J. Pediatr. Gastroenterol. Nutr. 12 (4), 501–506. 10.1097/00005176-199105000-00015 1678009

[B30] Kassim S. A. Abbas M. Tang W. Wu S. Meng Q. Zhang C. (2020). Retrospective study on melanosis coli as risk factor of colorectal neoplasm: A 3-year colonoscopic finding in zhuhai hospital, China. Int. J. Colorectal Dis. 35 (2), 213–222. 10.1007/s00384-019-03435-7 31823053

[B31] Katsumata R. Manabe N. Fujita M. Ayaki M. Sunago A. Kamada T. (2021). Colorectal neoplasms in melanosis coli: A survey in Japan and a worldwide meta-analysis. Int. J. Colorectal Dis. 36 (10), 2177–2188. 10.1007/s00384-021-03970-2 34156546

[B32] Kew S. T. Chakravarthi S. (2013). Images in clinical medicine: Melanosis coli. N. Engl. J. Med. 368 (24), 2303. 10.1056/NEJMicm1204882 23758235

[B33] Kim G. M. Jun E. J. Kim Y. C. Park J. M. Hong S. I. Cheung D. Y. (2011). Melanosis ilei induced by prolonged charcoal ingestion. J. Korean Surg. Soc. 81 (1), 66–69. 10.4174/jkss.2011.81.1.66 22066103 PMC3204554

[B34] Krbavcic A. Pecar S. Schara M. Müller K. Wiegrebe W. (1998). Anthranoid free radicals found in pseudomelanosis coli. Pharmazie 53 (5), 336–338. 9631503

[B35] Lestina L. S. (2001). An unusual case of melanosis coli. Gastrointest. Endosc. 54 (1), 119–121. 10.1067/mge.2001.115323 11427863

[B36] Li B. (2016). Advances in constituents and pharmacology of hedyotis diffusa. Tianjin Pharm. 28 (5), 75–78. 10.3969/j.issn.1006-5687.2016.05.027

[B37] Li C. Su X. Li F. Fu J. Wang H. Li B. (2019). Cytotoxic quinones from the aerial parts of Morinda umbellata L. Phytochemistry 167, 112096. 10.1016/j.phytochem.2019.112096 31470169

[B38] Li X. Zhou Y. Zhou S. Liu H. R. Xu J. M. Gao L. (2015). Histopathology of melanosis coli and determination of its associated genes by comparative analysis of expression microarrays. Mol. Med. Rep. 12 (4), 5807–5815. 10.3892/mmr.2015.4126 26238215 PMC4581826

[B39] Liang C. X. Wang S. S. Chen S. J. Wang Y. Li J. Chang Y. X. (2022). Research development on chemical composition and pharmacology of Polygoni Cuspidati Rhizoma et Radix. Chin. Tradit. Herb. Drugs 53 (04), 1264–1276.

[B40] Lin L. Ni B. Lin H. Zhang M. Li X. Yin X. (2015). Traditional usages, botany, phytochemistry, pharmacology and toxicology of polygonum multiflorum Thunb.: A review. J. Ethnopharmacol. 159, 158–183. 10.1016/j.jep.2014.11.009 25449462 PMC7127521

[B41] Liu J. Tian D. A. Wang J. P. Zhang S. Z. Feng J. Zhao Z. Z. (2011). Expression of aquaporin 8 and its relationship with melanosis coli. Chin. Med. J. 124 (19), 3061–3065. 22040556

[B43] Liu Z. H. Foo D. C. C. Law W. L. Chan F. S. Y. Fan J. K. M. Peng J. S. (2017). Melanosis coli: Harmless pigmentation? A case-control retrospective study of 657 cases. PLoS One 12 (10), e0186668. 10.1371/journal.pone.0186668 29088250 PMC5663380

[B44] Lombardi N. Bettiol A. Crescioli G. Maggini V. Gallo E. Sivelli F. (2020). Association between anthraquinone laxatives and colorectal cancer: Protocol for a systematic review and meta-analysis. Syst. Rev. 9 (1), 19. 10.1186/s13643-020-1280-5 31980030 PMC6979293

[B45] Lombardi N. Crescioli G. Maggini V. Bellezza R. Landi I. Bettiol A. (2022). Anthraquinone laxatives use and colorectal cancer: A systematic review and meta-analysis of observational studies. Phytother. Res. 36 (3), 1093–1102. 10.1002/ptr.7373 35040201 PMC9305424

[B46] Ma X. Q. Huang Q. Fu X. Y. Dong L. (2008). Comparison of the content of anthraquinone in Lycium barbarum L. From different regions and different years of ningxia. Lishizhen Med. Mat. Med. Res. (03), 636–637.

[B47] Malik E. M. Müller C. E. (2016). Anthraquinones as pharmacological tools and drugs. Med. Res. Rev. 36 (4), 705–748. 10.1002/med.21391 27111664

[B48] Mennecier D. Vergeau B. (2004). Melanosis coli? N. Engl. J. Med. 350 (2), 197; author reply 197. author reply 197. 10.1056/nejm200401083500223 14711927

[B49] Mohammed A. Paranji N. Singh A. Sanaka M. R. (2021). Pseudomelanosis coli, its relation to laxative use and association with colorectal neoplasms: A comprehensive review. JGH Open 5 (6), 643–646. 10.1002/jgh3.12546 34124379 PMC8171148

[B97] Mohtashami L. Amiri M. S. Ayati Z. Ramezani M. Jamialahmadi T. Emami S. A. (2021). Ethnobotanical uses, phytochemistry and pharmacology of different rheum species (Polygonaceae): a review. Adv. Exp. Med. Biol. 1308, 309–352. 10.1007/978-3-030-64872-5_22 33861453

[B50] Nekkaa A. Benaissa A. Mutelet F. Canabady-Rochelle L. (2021). Rhamnusalaternus plant: Extraction of bioactive fractions and evaluation of their pharmacological and phytochemical properties. Antioxidants 10 (2), 300. 10.3390/antiox10020300 33669348 PMC7920288

[B52] Nusko G. Schneider B. Schneider I. Wittekind C. Hahn E. G. (2000). Anthranoid laxative use is not a risk factor for colorectal neoplasia: Results of a prospective case control study. Gut 46 (5), 651–655. 10.1136/gut.46.5.651 10764708 PMC1727932

[B99] Oladeji O. S. Adelowo F. E. Oluyori A. P. Bankole D. T. (2020). Ethnobotanical description and biological activities of senna alata. Evid.-based Complement. Altern. Med. 2580259. 10.1155/2020/2580259 PMC7054808 32148534

[B98] Ou S. P. Ren L. Wang S. Chen L. Wang Y. H. (2017). Research on preparation and determination of Polygonum cuspidatum extract. J. Pharm. Res. 36 (10), 567–570+608. 10.13506/j.cnki.jpr.2017.10.003

[B53] Panichayupakaranant P. Sakunpak A. Sakunphueak A. (2009). Quantitative HPLC determination and extraction of anthraquinones in *Senna alata* leaves. J. Chromatogr. Sci. 47 (3), 197–200. 10.1093/chromsci/47.3.197 19298705

[B54] Peng C. Wang L. Wang Y. H. Li Y. X. Pan Y. (2009). The toxicity of aconitine, emodin on ICC cell and the antagonist effect of the compatibility. Eur. J. Drug Metabol. Pharmacokinet. 34 (3-4), 213–220. 10.1007/bf03191176 20166441

[B55] Peng X. B. Wang H. Yang T. (2012). Determination of anthraquinones content in rhizoma polygonati. J. Med. Pharm. Chin. Minor. 18 (8), 72–73. 10.3969/j.issn.1006-6810.2012.08.037

[B56] Qi L. Jiang H. Niu X. F. (2015). Study on the pharmacognosy of Polygonum suffultum var. pergracile. China J. Tradit. Chin. Med. Pharm. 5(17), 54–56+92.

[B57] Qian D. Zhao Y. Yang G. Huang L. (2017). Systematic review of chemical constituents in the genus Lycium (solanaceae). Molecules 22 (6), 911. 10.3390/molecules22060911 28629116 PMC6152755

[B58] Regitnig P. Denk H. (2000). Lack of Pseudomelanosis coli in colonic adenomas suggests different pathways of apoptotic bodies in normal and neoplastic colonic mucosa. Virchows Arch. 436 (6), 588–594. 10.1007/s004289900178 10917174

[B61] Rodríguez-Gómez I. M. Gómez-Laguna J. Ruedas-Torres I. Sánchez-Carvajal J. M. Garrido-Medina Á V. Roger-García G. (2021). Melanosis coli in pigs coincides with high sulfate content in drinking water. Vet. Pathol. 58 (3), 574–577. 10.1177/0300985821991565 33590812

[B62] Roerig J. L. Steffen K. J. Mitchell J. E. Zunker C. (2010). Laxative abuse: Epidemiology, diagnosis and management. Drugs 70 (12), 1487–1503. 10.2165/11898640-000000000-00000 20687617

[B100] Shan M. Yu S. Yan H. Chen P. Zhang L. Ding A. (2016). A review of the botany, phytochemistry, pharmacology and toxicology of rubiae radix et rhizoma. Molecules 21 (12). 10.3390/molecules21121747 PMC6274022 27999402

[B64] Shen Y. (2017). Sennoside A increases the risk of melanosis coli by inhibiting clostridia. Master. Nanjing Univ. Traditional Chin. Med.

[B66] Steer H. W. Colin-Jones D. G. (1975). Melanosis coli: Studies of the toxic effects of irritant purgatives. J. Pathol. 115 (4), 199–205. 10.1002/path.1711150403 1159566

[B67] van Gorkom B. A. Karrenbeld A. van der Sluis T. Zwart N. de Vries E. G. Kleibeuker J. H. (2001). Apoptosis induction by sennoside laxatives in man; escape from a protective mechanism during chronic sennoside use? J. Pathol. 194 (4), 493–499. 10.1002/path.914 11523059

[B68] Walker N. I. Bennett R. E. Axelsen R. A. (1988). Melanosis coli. A consequence of anthraquinone-induced apoptosis of colonic epithelial cells. Am. J. Pathol. 131 (3), 465–476. 3381879 PMC1880689

[B69] Walker N. I. Smith M. M. Smithers B. M. (1993). Ultrastructure of human melanosis coli with reference to its pathogenesis. Pathology 25 (2), 120–123. 10.3109/00313029309084783 8367190

[B70] Wan J. Z. Xu X. J. Guo H. X. Fang W. Liu Y. B. (2008). The researches on the coloring matter and the causes of the Melanosis coli and the relationship between Melanosis coli, Aloe and Rhizome. Int. J. Intern. Med. (04), 227–229+249.

[B71] Wang D. Wang X. H. Yu X. Cao F. Cai X. Chen P. (2021a). Pharmacokinetics of anthraquinones from medicinal plants. Front. Pharmacol. 12, 638993. 10.3389/fphar.2021.638993 33935728 PMC8082241

[B72] Wang J. (2012). Advances in the studies of Radix rubiae yunnanensis. Inn. Mong. J. Tradit. Chin. Med. 31 (23), 129–132. 10.16040/j.cnki.cn15-1101.2012.23.053

[B73] Wang J. F. Wang J. X. Teng Y. Wang S. S. Liu Y. (2017a). Determination of anthraquinones in Rumex acetosa L. J. Southwest Univ. Nat. Sci. Ed. 39 (04), 199–204. 10.13718/j.cnki.xdzk.2017.04.030

[B74] Wang K. Gui M. Y. Zhao P. F. Li J. Y. (2012). Impact of different processing methods on anthraquinones in aloe. Jiangsu Agric. Sci. 40 (8), 247–248. 10.3969/j.issn.1002-1302.2012.08.098

[B76] Wang S. Wang Z. Peng L. Zhang X. Li J. Yang Y. (2018). Gender, age, and concomitant diseases of melanosis coli in China: A multicenter study of 6,090 cases. PeerJ 6, e4483. 10.7717/peerj.4483 29568709 PMC5845562

[B77] Wang X. X. Wang C. J. Li Z. K. (2021b). Research progress on chemical composition, pharmacological activity of paederia scandens (lour.) merr. World Chin. Med. 16 (05), 826–830.

[B78] Wang Y. Huang L. L. Wang D. H. Fang F. Lai J. X. Xing C. C. (2017b). Research progress on chemical composition and function of plants of hemerocallis. Res. Pract. Chin. Med. 31 (01), 79–86. 10.13728/j.1673-6427.2017.01.022

[B79] Wei H. Y. Xie H. Liao Y. Dai P. (2019). Determination of total anthraquinone in saqgentodoxae Caulis and its antioxidant activity *in vitro* . Acta Med. Sin. 32 (04), 27–31. 10.19296/j.cnki.1008-2409.2019-04-008

[B80] Wei Y. L. Liang F. Tang H. M. (2011). Determination of total anthraquinones content in Rubia cordifolia. Guizhou Agric. Sci. 39 (08), 51–53.

[B81] Wilberts B. L. Schwartz K. J. Gauger P. C. Wang C. Burrough E. R. (2015). Evidence of oxidative injury in pigs with melanosis coli. Vet. Pathol. 52 (4), 663–667. 10.1177/0300985814559403 25421421

[B83] Xu L. Chan C. O. Lau C. C. Yu Z. Mok D. K. Chen S. (2012). Simultaneous determination of eight anthraquinones in Semen Cassiae by HPLC-DAD. Phytochem. Anal. 23 (2), 110–116. 10.1002/pca.1331 21618311

[B84] Yadav J. P. Arya V. Yadav S. Panghal M. Kumar S. Dhankhar S. (2010). Cassia occidentalis L.: A review on its ethnobotany, phytochemical and pharmacological profile. Fitoterapia 81 (4), 223–230. 10.1016/j.fitote.2009.09.008 19796670

[B87] Yang N. Ruan M. Jin S. (2020a). Melanosis coli: A comprehensive review. Gastroenterol. Hepatol. 43 (5), 266–272. 10.1016/j.gastrohep.2020.01.002 32094046

[B88] Yang N. Ruan M. Jin S. (2020b). Melanosis coli: A comprehensive review. Gastroenterol. Hepatol. 43 (5), 266–272. 10.1016/j.gastrohep.2020.01.002 32094046

[B90] Younes M. Aggett P. Aguilar F. Crebelli R. Filipič M. Frutos M. J. (2018). Safety of hydroxyanthracene derivatives for use in food. Efsa J. 16 (1), e05090. 10.2903/j.efsa.2018.5090 32625659 PMC7009633

[B91] Zhang C. H. Yang X. Wei J. R. Chen N. M. Xu J. P. Bi Y. Q. (2021a). Ethnopharmacology, phytochemistry, pharmacology, toxicology and clinical applications of Radix Astragali. Chin. J. Integr. Med. 27 (3), 229–240. 10.1007/s11655-019-3032-8 31502185

[B92] Zhang W. Sun C. Zhou S. Zhao W. Wang L. Sheng L. (2021b). Recent advances in chemistry and bioactivity of Sargentodoxa cuneata. J. Ethnopharmacol. 270, 113840. 10.1016/j.jep.2021.113840 33460761

[B93] Zhang Y. (2013). The study of effects of anthraquinone herbs on melanosis coli. Master. Fourth Mil. Med. Univ.

[B95] Zhao M. Duan J. A. Huang W. Z. Zhou R. H. Che Z. T. (2003). Steroids and anthraquinones from Astragalus hoantchy. J. China Pharm. Univ. (03), 22–25.

[B96] Zheng Z. Z. (2017). *Study on Metabolism and Toxicity of Alizarin and Pharmacokinetics of ethanol Extract of madder.* Master. Guangzhou: Traditional Chinese Medicine University Of Guangzhou.

